# Supplementary material for: The role of grammar in transition-probabilities of subsequent words in English text
Source: PLoS One. 2020 Oct 8;15(10):e0240018. doi: 10.1371/journal.pone.0240018 (PMC7544142; doi:10.1371/journal.pone.0240018)
Supplement: Attachment — Submitted filename: PONE-D-20-13476R1 R2R.docx [file pone.0240018.s001.docx]

General comment:

We added a whole paragraph in the manuscript discussing in detail the aim and scope

of the paper. We have done so to help the reader to correctly interpret the presented

results and the conclusions that can be drawn from them.

**Response to the Reviewer:**

1. At several points, the text is unacceptably imprecise. It sometimes makes the

impression that the authors didn't give it a thorough reading before submission.

For instance:

_ We are told that Fig. 1 shows data for "The Two Captains", but the _gure

caption says otherwise.

Response: Thank you for seeing this. We have corrected this faulty reference

to _gure 1 in the text.

_ In section 0.1 (?!), we read

"Imagine the _rst word in a sentence is randomly drawn from the entire

lexicon with W words (states), say "The wolf". As soon as it is decided, the

second word must be a verb".

First, "The wolf" are two words, not one. Second, it is not true that grammar

imposes that the next word is a verb. I can imagine in_nitely many sentences

where the next word is "of", "in", "that", "however", "therefore", etc. etc.

etc.

**Response**: Thank you for pointing this out. We have adapted this passage

accordingly by expanding the discussion on constraints. We have taken pains

to clean this kind of imprecisions in the manuscript. In the particular case

we now are speaking of the `_rst word group' instead of the `_rst word', for

instance. We also discuss in more detail how a sentence, out of context,

does not feel many constraints and therefore, as you point out, could take

any shape and could be continued in quite arbitrary ways. We included

a note that before the second word group (the verb-group) starts the _rst

group \The wolf" could be further elaborated, e.g. "The wolf with the

red fur". We also state more strongly than we did that it are primarily

contextual constraints that are governing word choices, which in our model

are represented by the SSR word selection process, and that, in comparison

to those globally acting constraints, grammatical constraints act locally on

those choices. We have over and all tried to streamline the logic underlying

the paper and make it more transparent.

_ The labels S, P and O, used at various points in the text, are never de_ned.

Response: Thank you for pointing out that those labels may not have been

su_ciently characterized. Those labels are now de_ned, (a) loosely as a

primitive subject-predicat-object labelling of words in very simple English

sentences such as \Peter{watches{clouds." and (b) as a symbolic notation

for the case of three abstract grammatical labels S=1, P=2, and O=3 in

the toy grammar we use to augment SSR processes and obtain goSSR word

sequences. We believe that, at the level of the statistical analysis used in the

paper, a more concrete de_nition of those labels is not indicated, due to the

toy character of the grammar used in the model.

_ At the beginning of section 1, we are told that the analysis concerns three

novels. However, "Five Little Peppers" is rather a series of novels, and the

"Collected Tales by the Brothers Grimm" is -quite obviously- a collection of

tales, not a novel.

**Response**: Thank you for pointing this out. We list the titles of six texts

(we have added three additional texts to the analysis/exempli_cation of the

principle the paper explores) and indicate particular types of texts, such as

collections of tales or part of a book series. When referring to those works in

the body of the manuscript we now neutrally refer to all of them as `text'.

These are just four examples of a long series of presentation inconsistencies that

the authors must carefully consider and solve in a revision.

Response: We have taken pains to detect such inconsistencies and resolve them,

including the one or other `lost word' we found. We also removed a sentence

that referred to an older version of a _gure and had become obsolete. We have

tried to improve the overall coherence of the manuscript and tried to make several

passages of the manuscript clearer. Also we added additional discussions where

those seemed indicated or resolved some other smaller issues we noted.

2. In the statistical analysis of language, it is not good practice to work with

translated texts. The e_ects of translation on the statistics of word usage, vocabulary

choice, grammatical correlations, and so on, have never been assessed, and depend

upon a series of more or less uncontrolled factors. Just think of the quality of

translation. Statistical measurements on translated texts might be biased by

elements whose characterization would need a fully separate -not yet undertaken-

study. Unfortunately, of a total of three works, the present results concern two

translations. I urge the authors to redo their analysis for texts in their original

language. It would also help interpreting the results if the works have similar

lengths and vocabulary sizes.

**Response**: Thank you. We had not fully realized that also `Two captains' is

a translation from German into English. We agree completely that a in depth

analysis of the issue would in fact require to group texts into sub-categories of

comparable length and maybe other parameters, such as the time the text was

written. In particular this were true if the aim of the paper were for instance to

evaluate how the model parameter Ng behaves in distinct subclasses. However,

we have a much more modest aim, as we now precisely state and discuss in

the manuscript, namely to show that a grammatical ordering process can explain

why word-transition frequencies of real texts resemble those of a Bernoulli process

rather than that of structured word selection processes, as for instance provide by

the SSR model (but in principle also by the Simon model). The conclusions the

paper draws do not require more than a demonstration of the e_ect grammatical

ordering has on an underlying word selection process and its robustness with

respect to type and size of text. In order to demonstrate the e_ect of augmenting

a word selection process with a grammatical ordering process and its robustness,

exempli_cation of the e_ect in a few more or less randomly picked texts that

cover various text lengths and types therefore seems adequate and su_cient, in

particular as an in-depth analysis of gets restricted by computational constraints.

We fully agree that three examples were clearly insu_cient, in particular because

two were translations. We have therefore added three more texts that are original

English texts and not translated from another Language. We discuss the choice

we make (exempli_cation over in-depth analysis), in a new paragraph added to

the introduction of the manuscript where we also state clearly the basic aim of

the paper. Other paragraphs that already existed in the previous version of the

manuscript and touch this issue you raise here, have also been updated, aiming

for a higher precision and level of clarity of the manuscript, mainly by clearly

pointing out the comparably modest aim of the paper.

3. A major drawback of the model of text generation presented in this manuscript

is that the SSR process acts at the level of sentences only. Namely, the process

is renewed at the beginning of each sentence. However -as has been discussed

at length in connection with Simon's model for Zipf's law- the e_ect of word

choices extends over much longer scales, at the level of full chapters and even the

entire work. The reason for this is, of course, evident: the initial choice of words

establishes the semantic context of the full work -concretely, what the work tells

us about- and therefore strongly constrains the appearance of new words over the

whole text.

This point is completely overlooked in the manuscript. By itself, such failure

does not invalidate the contribution. However, the point is so obvious that it is

mandatory, for the sake of scienti_c correctness, that the authors comment on it

in su_cient detail. It would be welcome, in fact, that they discuss how the model

could be extended to encompass longer contextual spans.

**Response**: Thank you for pointing this out. We have added a paragraph in

the introduction section discussing this point. We state more clearly now that

constraints are mainly context related and therefore those constraint are not

restricted to the sentence level but rather act on possibly all scales of a text.

In the model this globally acting constraints are implicitly represented by the

SSR word selection process. We also state explicitly that the restarting of word

selection sequences are not in principle bound to the sentence level but could

also work on the level of paragraphs or sections. We point out that we restart

the sequences with every sentence for reasons of model simplicity. Our results,

however, would not di_er qualitatively from the presented ones if we were to

restart word selection sequences after a number of sentences, where this number

gets sampled from a Poisson distribution, for instance. The grammatical ordering

scheme would still scramble word sequences locally and thus make them resemble

samples of respective Bernoulli sequences more closely, which is what we want

to demonstrate in the paper. Such an analysis would again need to be part of a

in-depth analysis that one would have to perform in order to optimize and gage

the model for exploring properties of real texts that could be detected by _tting

respective model parameters, such as `grammatical depth/complexity'. We point

out in the manuscript that in-depth analysis of this kind goes beyond the scope

and the aim of this paper.

We hope that the reviewer is similarly happy with the improvement of the manuscript

as we are. Thank you for your efforts.
